# Effects of Shot Peening Pressure, Time, and Material on the Properties of Carburized Steel Shafts

**DOI:** 10.3390/ma17164124

**Published:** 2024-08-20

**Authors:** Shao-Quan Lu, Liu-Ho Chiu, Pei-Jung Chang, Chung-Kwei Lin

**Affiliations:** 1 Department of Mechanical and Materials Engineering, Tatung University, Taipei 104-327, Taiwan; d11101001@o365.ttu.edu.tw; 2 Research Center of Digital Oral Science and Technology, College of Oral Medicine, Taipei Medical University, Taipei 110-301, Taiwan; peronchang@tmu.edu.tw; 3 Graduate Institute of Manufacturing Technology, National Taipei University of Technology, Taipei 106-344, Taiwan; 4 School of Dental Technology, College of Oral Medicine, Taipei Medical University, Taipei 110-301, Taiwan

**Keywords:** carburized steel, shot peening, residual stress, retained austenite, surface roughness

## Abstract

Carburized steel shafts are commonly used in industry due to their good wear resistance and fatigue life. If the surface of carburized shafts exhibits an undesired tensile stress, shot peening treatment may be required to alter the stress condition on the surface. In the present study, the effects of shot peening pressure (3–5 kg/cm^2^), time (32–64 s), and material (stainless steel, carbon steel, and glass) on the residual stress, retained austenite, microhardness, and surface roughness of the carburized shafts were investigated. The experimental results showed that the surface residual tensile stress was changed into compressive stress after the shot peening treatment. The shot peening effects increased with the increasing peening pressure and time. In addition, a significant decrease in the amount of retained austenite in the subsurface region was observed. Peening with different materials can affect the peening effect. Using glass pellets exhibited the best shot peening effect but suffered massive pellet fracture during processing. In overall consideration, the optimal peening parameters for carburized steel shafts for practical industrial applications involved using the stainless-steel pellets with a peening pressure of 5 kg/cm^2^ and a peening time of 64 s. The maximum residual stress was −779 MPa at a depth of 0.02 mm, while the highest surface microhardness was 827 HV_0.1_.

## 1. Introduction

Mechanical parts used in industry are usually required to possess good wear resistance and fatigue life to ensure their proper function. A major factor that affects these properties is the strength of the materials used to manufacture these mechanical parts. Other factors that affect resistance to mechanical damage include heat treatment procedures and surface treatments. When selecting materials for mechanical parts, additional properties, such as workability during machining, toughness in practical usage, and reasonable manufacturing costs, are also considered. For mechanical parts, such as shafts and gears, steel materials are a popular choice due to their strength in resisting wear and fatigue. Among them is low-carbon alloy steel, which is considered one of the most promising materials.

The carburizing treatment of low-carbon steel is often used to strengthen the surface of mechanical parts. Carburizing treatment can not only increase the surface hardness of mechanical parts but can also affect changes in microstructures on the subsurface region. After carburizing, the toughness of the core remains [1,2,3], whereas a higher surface hardness can provide superior wear resistance. The carburizing treatment, however, increases the carbon concentration, inducing formation of retained austenite. Research by Rivero et al. [4] mentioned that a high content of retained austenite in the subsurface can be observed after the carburizing treatment. The retained austenite is a metastable phase and can be easily transformed into martensite by external environmental factors, such as temperature and load. This affects the stability of low-carbon steel mechanical parts in subsequent usage and may result in a poor fatigue life. Roy et al. [5] reported that the retained austenite on the surface for carburized steel will induce phase transformations under the stress conditions, which result in a shortened fatigue life.

To resolve the retained austenite issue mentioned above, cryogenic treatment and shot peening are commonly used processes in the industry. Cryogenic treatment is relatively expensive and used for carburized steel parts of a unique geometry. The shot peening treatment of carburized low-carbon steel parts is generally the priority choice because it can result in a compressive residual stress on the subsurface regions and greatly improve the fatigue life. Residual stress is an important factor affecting the fatigue life of carburized steel parts [6,7,8,9]. The shot peening process has low geometric restrictions, and the results are usually the most economical and highly effective. Shot peening is often used in the surface-strengthening process of mechanical parts. Plastic deformations occur during the process and increase the hardness in the subsurface region due to work hardening. In addition, uniform residual compressive stress can be observed after the shot peening. For instance, Lin et al. [10], Ongtrakulkij et al. [11], and Kikuchi et al. [12] conducted relevant research on the distribution of residual compressive stress on the surface after shot peening. They confirmed that the shot peening treatment of aluminum alloys [10], titanium alloys [11], and steel [12] will induce the conversion of residual stress from a tensile stress to a compressive stress state.

Various processing parameters, especially the shot peening intensity and the coverage, can affect the effectiveness of the shot peening treatment. For instance, the higher the peening intensity is, the larger the compressive residual stress [13,14]. In addition, the duration time of the shot peening process also affects the compressive residual stress. Typically, a maximum coverage of 200% (the time required to shot peen the whole area twice) is commonly used in industry [15,16]. Wu et al. [17] reported that the 200% coverage of shot peening treatment significantly increased the compressive residual stress and the hardness in the subsurface area. Lin et al. [18] discussed the effects of speed and coverage of shot peening. Their results showed that increasing the intensity of shot peening can increase the depth of the residual compressive stress. In addition, the maximum residual compressive stress value increased with the increasing duration time of the shot peening process. Qu et al. [19] and Song et al. [20] showed that increasing the shot peening coverage to 200% can refine the grain size of martensite, increase the hardness, and increase the residual compressive stress. As addressed above, verification after the shot peening treatment typically examines the residual stress state and hardness. To date, most of the studies investigated the relationship between the shot peening parameters (intensity and coverage) and the resulting properties (residual stress and hardness). However, there is a lack of papers addressing the effects of different shot peening parameters on the residual stress and retained austenite content of low-carbon alloy steel carburized parts. In addition, few studies have reported the effects of different peening materials on the properties of carburized steel.

In the present study, a bicycle shaft is the target material to be investigated. The shaft is used to connect the crank and frame of a bicycle, and thus experiences high-torsion force and severe wear during usage. The steel shaft was required to undergo appropriate surface modification to extend its service life in practical application. The steel shafts were first carburized to achieve suitable surface hardness. The carburized shafts were then shot-peened with different parameters, including peening pressure, time (i.e., coverage), and materials. The residual stress, amount of retained austenite, hardness, and surface roughness of the shot-peened carburized shafts were examined to determine the effects of shot peening treatment. The optimal shot peening parameters for a carburized shaft were suggested for practical industrial applications.

## 2. Materials and Methods

### 2.1. Carburization of Steel Shafts

**Figure 1:**
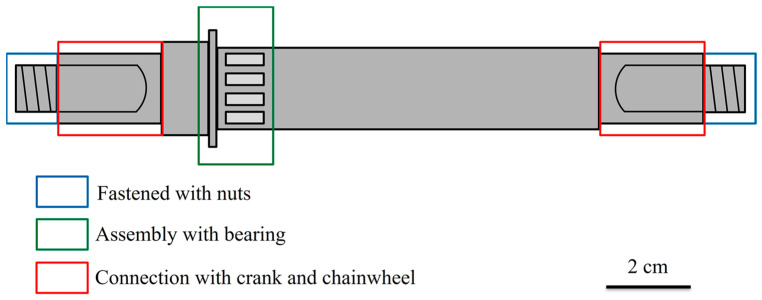
Schematic illustration of the bicycle shaft used in the present study. The red rectangle areas are the shot peening areas.

**Figure 2:**
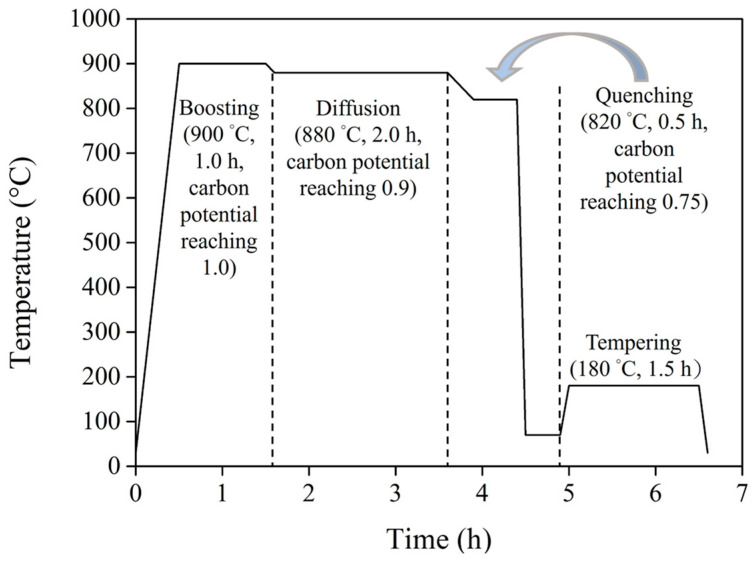
The schematic illustration of the carburizing heat treatment process.

The specimens used in the experiment were the bottom bracket shafts of a bicycle, as shown in Figure 1. The shaft was made of JIS SCM415, a low-carbon alloy steel manufactured by China Steel Corporation (Kaohsiung, Taiwan), and Table 1 lists its chemical compositions. The shafts were surface-hardened by carburizing heat treatment according to the procedures shown in Figure 2. First (boosting stage), the shafts were heated from room temperature to 900 °C at a heating rate of 22 °C/min and exposed to an atmosphere with a carbon potential of 1.0% for 1 h. The carbon potential was the carbon atom concentrations (provided by CO gas) in the atmosphere of the heat treatment furnace, at which a high concentration of carbon atoms was diffused into the surface layer of the low-carbon steel shaft. Then, the temperature was slightly decreased to 880 °C and held for 2 h in an atmosphere with a carbon potential of 0.9% (diffusion stage). After the carburizing process (boosting and diffusion), the temperature was decreased to 820 °C under an atmosphere with a carbon potential of 0.75% for another 0.5 h and then oil-quenched to 70 °C in 30 s (quenching stage). In the final stage, the carburized shafts were tempered at 180 °C for 1 h and cooled with water.

The carburizing heat treatment was confirmed by examining the surface hardness and the effective case depth. The surface hardness of the carburized shaft was 60 ± 2 HRC (HRC, FUTURE-TECH FR-3e, Kawasaki, Japan) with an applied load of 150 kg. The effective case depth was examined using a Vickers microhardness tester (HV, Matsuzawa MXT50, Akita, Japan) with an applied load of 300 g. The effective case depth was determined by examining the depth with a microhardness of 550 HV and was 0.5 ± 0.02 mm.

### 2.2. Shot Peening of Carburized Shafts

The carburized shafts were followed by shot peening treatment, for which the shot peening pressure (intensity), shot peening time (coverage), and peening pellets with different materials were selected to perform shot peening on the surface of the carburized shafts. The shot peening machine used in the experiment was a homemade system that included a contained box, an automatic air compressor, and a circled feeding system. The nozzle diameter was 4 mm and the nozzle moving speed was set to 4 mm/s. The distance from the nozzle to the shaft was 100 mm. The ink tracer method [16] was used to measure the coverage of the shot peening treatment, and it took 32 s for coverage to reach 100%. The peening materials used in the present study included stainless-steel pellets (NR 0.3, SUS304), carbon steel pellets (TS-30, close to JIS S45C), and glass pellets (B60, SiO_2_). All the pellets were purchased from Rich Sou Technology Co., Ltd. (Kaohsiung, Taiwan). Table 2 summarizes all the shot peening parameters and coded peening materials.

### 2.3. Characterizations of Shot-Peened Carburized Shafts

After the shot peening treatment, the residual stress, the amount of retained austenite, the microhardness, and the surface roughness of the shot-peened carburized shafts were measured and addressed, as follows.

#### 2.3.1. X-ray Diffraction

The residual stress and the amount of retained austenite were measured with a portable X-ray diffractometer (Pulstec μ-X360s, Pulstec Industrial Co., Ltd., Shizuoka, Japan), applying the measuring principle of the single-incident-angle method (cos*α* method) [21,22,23]. Firstly, surface residual stress and the amount of retained austenite were examined. Each specimen was measured three times at different locations around the middle of the carburized shafts. Afterwards, surface material was removed by electropolishing up to a maximum depth of 0.2 mm and in-depth measurements were performed as a function of depth. The μ-X360s used a Cr target that was operated at a voltage of 30 kV and a current of 1 mA, with a diffraction angle of 35° for residual stress and 0° for retained austenite analysis. The X-ray diffraction parameters are listed in Table 3. The alignment of the X-ray diffractometer was confirmed by using a stress-free iron powder calibration sample prior to the experiments. The amount of retained austenite (γ %) in the microstructure was calculated using the equation shown in Equation (1) [24]. Iγ and Iα are the integrated intensity for austenite and ferrite, respectively. Rγ and Rα are the theoretical relative intensities for austenite and ferrite, respectively.

γ % = (Iγ/Rγ)/[(Iγ/Rγ) + (Iα/Rα)] × 100%(1)

#### 2.3.2. Microhardness

To determine the effect of different shot peening parameters on hardness in the subsurface region, a Vickers hardness tester (Matsuzawa MXT50, Akita, Japan) was used to measure the microhardness (HV) values, from the surface to a depth of 0.2 mm at an interval of 0.01 mm. The microhardness (*n* = 5) at various depths was measured with an applied load of 100 g (0.98 N) for a duration of 10 s. A microhardness curve was established in the subsurface region to compare the effects of shot peening treatments with different parameters.

#### 2.3.3. Surface Roughness

For the surface roughness (Ra) measurement of the shot-peened shaft, a surface roughness analyzer (Mitutoyo SV-400, Kanagawa, Japan) was used to measure the surface roughness values three times at different locations around the middle of the carburized shafts before and after the shot peening treatments.

## 3. Results and Discussion

### 3.1. The Effect of Shot Peening and Peening Pressures on the Carburized Shaft

**Figure 3:**
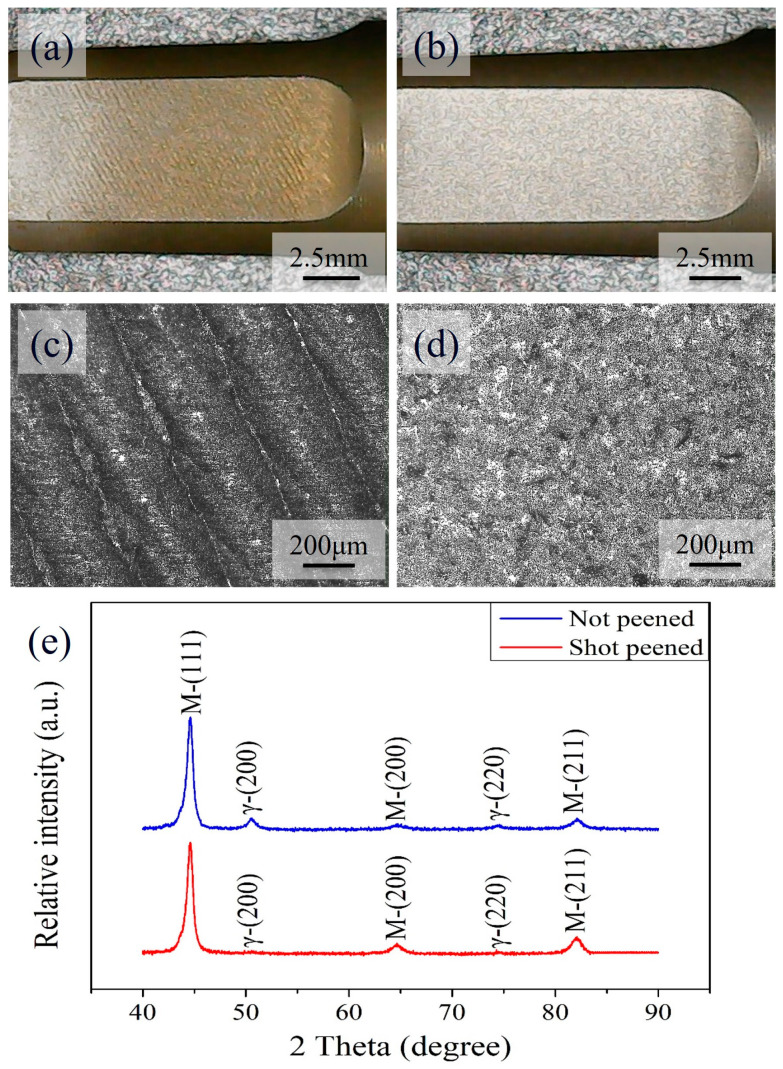
Surface photos of the carburized shaft (**a**) before and (**b**) after shot peening treatment. Corresponding optical microscope images are shown in (**c**,**d**), whereas the X-ray diffraction patterns are shown in (**e**). The shot peening areas are the connections between the crank and chainwheel, the red rectangle areas in Figure 1.

Figure 3 shows the preliminary characterization of the shaft after the shot peening treatment. Figure 3a,b show photos of the flat surface before and after the shot peening treatment. Several machining traces before peening (Figure 3a) disappeared after the treatment (Figure 3b). This can be better observed under an optical microscope, and Figure 3c,d show the corresponding images, respectively. In addition, conventional X-ray diffraction was used to examine the peened area, and the XRD patterns are shown in Figure 3e. It can be noted that, before peening treatment, the carburized shaft exhibited a major martensitic phase (PDF No. 44-1290) with minor retained austenite (PDF No. 65-4150). After peening treatment, partially retained austenite phase was transformed into martensite. This was revealed by the decrease in the peak intensities of austenite, shown by the bottom red curve in Figure 3e, at 50.01° and 73.4° for γ(200) and γ(220), respectively. Meanwhile, a slight peak broadening and an increase in the peak intensities of martensite were observed, as shown in Figure 3e. Magnified SEM images and XRD patterns are available in Figures S1 and S2, respectively.

**Figure 4:**
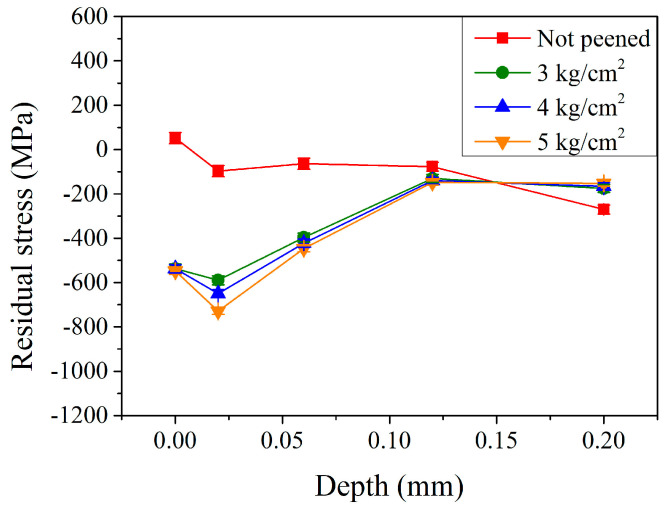
Residual stress profiles of carburized shafts with different peening pressures.

To further examine the condition of residual stress and the amount of retained austenite, a portable X-ray diffractometer, applying the measuring principle of the single-incident-angle method (cos*α* method), was used [21,22,23]. As shown by the red curve in Figure 4, the residual stress on the surface of the un-peened carburized shaft was 52 MPa. With increasing the depth to 0.02 mm, the stress state changed from a tensile stress to a compressive stress of −97 MPa. It slightly varied to −64 and −77 MPa at a depth of 0.06 and 0.12 mm, respectively. Then, it significantly decreased to −270 MPa at a depth of 0.20 mm. It should be pointed out that martensitic transformation was accompanied by volume expansion, which induced compressive stress. However, the surface residual stress appeared as tensile residual stress due to the boundary effect, whereas compressive residual stress persisted within the interior of the carburized layer. After the shot peening, the residual stress within the carburized shaft changed significantly. As shown by the green curve in Figure 4, the surface residual stress changed from a tensile stress of 52 MPa to a compressive stress of −537 MPa with a peening pressure of 3 kg/cm^2^. It decreased to a maximum compressive residual stress of −590 MPa at a depth of 0.02 mm. It then increased continuously to −397 and −130 MPa at a depth of 0.06 and 0.12 mm, respectively. Thereafter, it slightly increased to −175 MPa at a depth of 0.20 mm. A similar trend was observed with higher peening pressures at 4 and 5 kg/cm^2^ (the blue and orange curves in Figure 4, respectively). A major difference was observed at a relatively lower depth of 0.02 mm, where the maximum residual compressive stress was −590, −652, and −729 MPa, with a peening pressure of 3, 4, and 5 kg/cm^2^, respectively. Shot peening effectively made the residual compressive stress distributed on the surface of parts [18,19]. In this study, the maximum residual compressive stress observed at a depth of 0.02 mm was probably due to the relatively small size of the pellets (0.3 mm) [18]. The effect of peening pressure (3–5 kg/cm^2^) was similar at the surface of the carburized shaft. This shows a similar trend to that reported by Kikuchi et al. [12] and Chen et al. [13]. The maximum residual compressive stress value generated in the subsurface region, however, was affected by the peening pressure. The stress increased with the increasing peening pressure from −590 MPa (3 kg/cm^2^) to −729 MPa (5 kg/cm^2^), which are results similar to that reported by Song et. al. [20].

**Figure 5:**
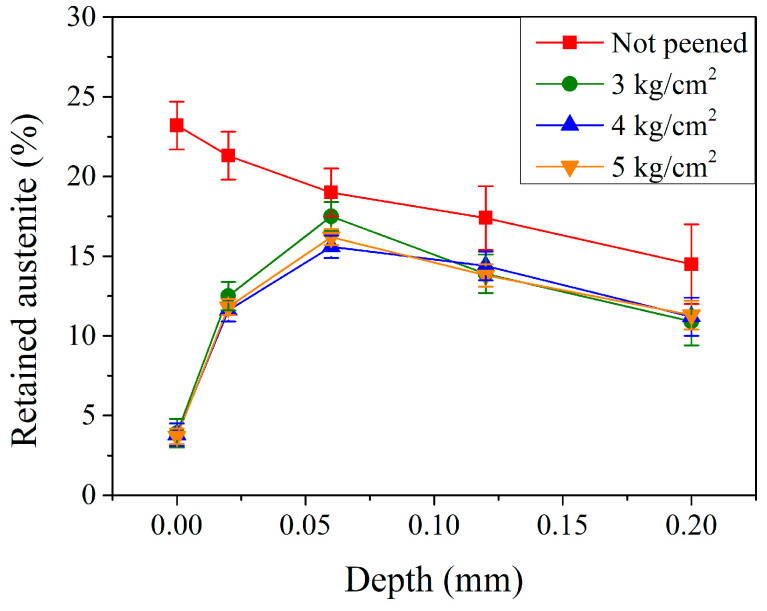
Retained austenite profiles of carburized shafts with different peening pressures.

Figure 5 shows the amount of retained austenite in the subsurface region of the carburized shaft before and after the peening treatment. For the un-peened carburized shaft (the red curve in Figure 5), the retained austenite content on the surface was 23.2%. It decreased continuously with increasing depth and was 21.3, 19.0, 17.4, and 14.5% at 0.02, 0.06, 0.12, and 0.20 mm, respectively. After the shot peening, the retained austenite content within the carburized shaft changed significantly. As shown by the green curve in Figure 5, the retained austenite on the surface decreased from 23.2% (un-peened shaft) to 3.9% (the minimum) with a peening pressure of 3 kg/cm^2^. It increased to 12.5% at a depth of 0.02 mm and reached a maximum of 17.5% at 0.06 mm. Thereafter, it decreased gradually to 13.9 and 10.9% at depths of 0.12 and 0.20 mm, respectively. A similar trend was observed when higher peening pressures of 4 and 5 kg/cm^2^ (the blue and orange curves in Figure 5, respectively) were used. A major observed difference was the maximum retained austenite at a relatively low depth of 0.06 mm, where the retained austenite was 16.0 and 15.6% with peening pressures of 4 and 5 kg/cm^2^, respectively. This was smaller than that (17.5%) treated with a peening pressure of 3 kg/cm^2^. Roy et al. [5] reported that the retained austenite can transform into martensite via external environmental factors, such as temperature and load. With the energy input from the shot peening treatment, the retained austenite was transformed into martensite. After the shot peening treatment, the retained austenite content was significantly reduced to a minimum on the surface. They were 3.9, 3.8, and 3.7% without significant differences when peening with pressures of 3, 4, and 5 kg/cm^2^, respectively. The retained austenite content reached a maximum in the subsurface region at a depth of 0.06 mm, and they were 17.5, 16.0, and 15.6% with peening pressures of 3, 4, and 5 kg/cm^2^, respectively. The higher the peening pressure, the smaller the maximum retained austenite.

**Figure 6:**
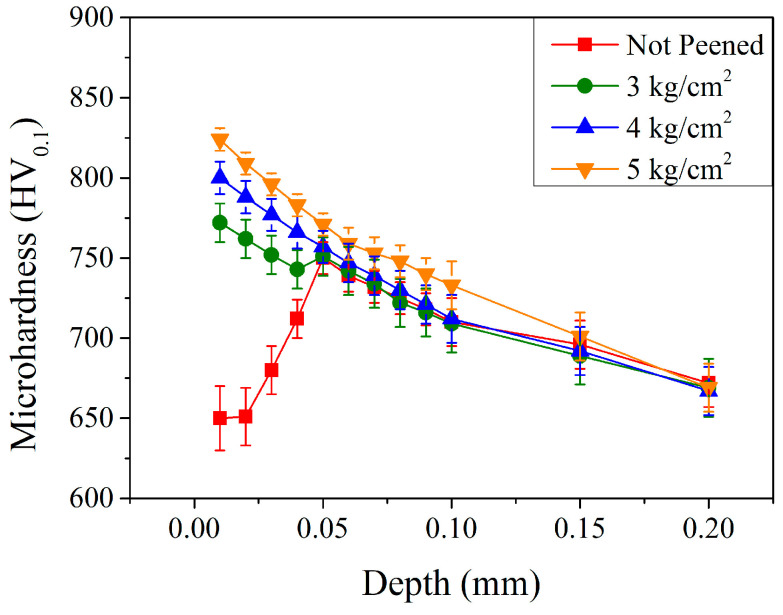
Microhardness profiles of carburized shafts with different peening pressures.

The mechanical property of the carburized shaft as a function of depth was investigated using the Vickers microhardness test with an applied load of 100 g. Before the peening (the red curve in Figure 6), the microhardness was ~650 HV_0.1_ at relatively shallow depths of 0.01 and 0.02 mm. It increased significantly to 680 HV_0.1_ at a depth of 0.03 mm and increased continuously to a maximum of 751 HV_0.1_ at 0.05 mm. Then, it decreased gradually to 672 HV_0.1_ at a depth of 0.20 mm. The microhardness of the un-peened shaft was basically affected by the carburization and quenching treatment. Though the carburized treatment induced a carbon concentration gradient due to the diffusion effect through the shaft, the descending carbon content was expected to result in a descending microhardness. The subsurface layer (up to 0.05 mm), however, exhibited a larger amount of retained austenite (recalled Figure 5) and resulted in an ascending microhardness. The microhardness profile was similar to those reported in the literature [25,26,27,28,29]. After the shot peening, the microhardness increased significantly in the subsurface region up to a depth of 0.05 mm. As a general trend, the microhardness of the shaft increased with the increasing peening pressure. The microhardness exhibited a maximum at a depth of 0.01 mm and was 772, 800, and 824 HV_0.1_ with peening pressures of 3, 4, and 5 kg/cm^2^, respectively. The microhardness increase in the subsurface region can be attributed to the phase transformation of a relatively soft retained austenite into a hardened martensite. It decreased continuously to 669, 667, and 669 HV_0.1_ at a depth of 0.20 mm. Lin et al. [18] and Qu et al. [19] also reported a similar trend for the increase in microhardness in the subsurface region with the increase of the peening pressure. The increase in peening pressure increased the energy transferred into the carburized shaft and resulted in an increase in microhardness, mainly due to the retained austenite–martensite transformation.

### 3.2. The Effect of Peening Time

**Figure 7:**
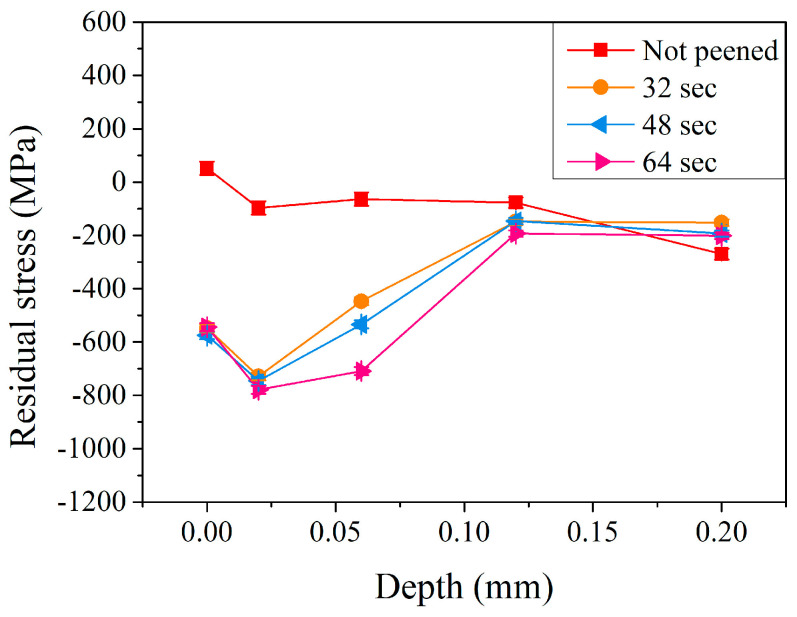
Residual stress profiles of carburized shafts after the peening treatment at a peening pressure of 5 kg/cm^2^ and different peening times.

In Section 3.1, the effects of peening and peening pressure were investigated. The higher the peening pressure, the higher the residual stress and microhardness. A peening pressure of 5 kg/cm^2^ (with a duration of 32 s) was used to further examine the effect of peening time. Figure 7 shows the residual stress distribution in the subsurface region of the carburized shaft before and after the peening treatment using a pressure of 5 kg/cm^2^ with various peening times (32, 48, and 64 s). A similar trend was observed with different peening times. Residual compressive stress within the carburized shaft increased significantly, especially at a depth of 0.06 mm. At a relatively short peening time (32 s, the orange curve in Figure 7), the surface residual stress was 52 MPa before peening and significantly changed to a compressive stress of −548 MPa after peening for 32 s. It decreased to a maximum compressive residual stress of −729 MPa at a depth of 0.02 mm and increased continuously to −447 and −148 MPa at a depth of 0.06 and 0.12 mm, respectively. The residual stress was −152 MPa at a depth of 0.20 mm. Thus, increasing the peening time resulted in a higher residual stress. At a depth of 0.06 mm, the residual compressive stress was −447, −534, and −709 MPa with peening times of 32, 48, and 64 s, respectively. Similar behavior was reported by Lin et al. [10,18] and Wu et al. [17]. The extended peening time will increase the residual compressive stress in the deeper depths of the subsurface region.

**Figure 8:**
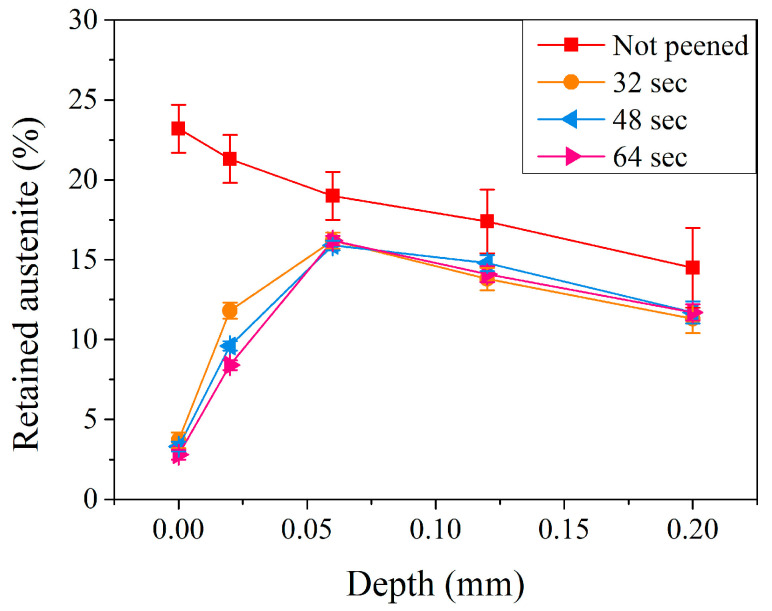
Retained austenite profiles of carburized shafts after the peening treatment at a peening pressure of 5 kg/cm^2^ and different peening times.

The amount of retained austenite in the subsurface region of the carburized shaft after the peening with a pressure of 5 kg/cm^2^ with different peening times was examined, and Figure 8 shows the corresponding results. Similar to that illustrated in Figure 6 (the effect of peening pressure with a 32 s peening time), the retained austenite decreased significantly at the surface, increased rapidly to a maximum, and decreased slightly thereafter. With a peening time of 32 s, the retained austenite decreased from 23.2% for the un-peened carburized shaft to 3.7%. It increased quickly to a maximum of 16.2% at 0.06 mm and decreased gradually to 11.3% at a depth of 0.20 mm. With prolonged peening treatment for a duration of 64 s, it can be noted that a major difference was exhibited at a depth of 0.02 mm, and the retained austenite was 11.8, 9.6, and 8.4% after peening times of 32, 48, and 64 s, respectively. The longer the peening time, the more energy input during the peening treatment, and the smaller the amount of retained austenite.

**Figure 9:**
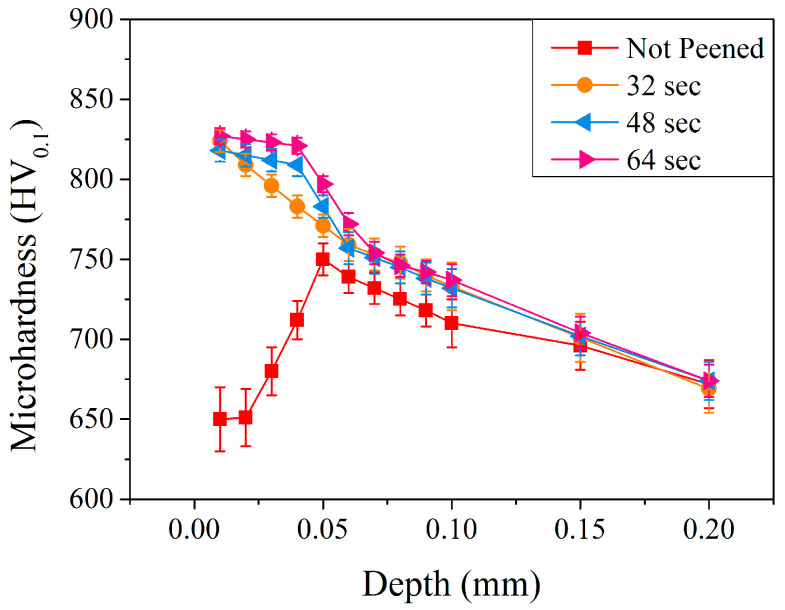
Microhardness profiles of carburized shafts after the peening treatment at a peening pressure of 5 kg/cm^2^ and different peening times.

Figure 9 shows the results of microhardness in the subsurface region of the carburized shaft. It can be noted that, for up to a depth of 0.06 mm, the longer the peening time, the higher the microhardness. Compared to those with a peening pressure of 5 kg/cm^2^ and a peening time of 32 s (the orange symbols), the microhardness remained at a relatively high value up to a depth of 0.04 mm when treated with a prolonged peening time. At a depth of 0.01 mm, the microhardness was 818 HV_0.1_ and 827 HV_0.1_ with peening times of 48 and 64 s, respectively. For a relatively short peening time of 32 s, it decreased significantly to 783 HV_0.1_. The microhardness, however, was 809 HV_0.1_ and 821 HV_0.1_ at a depth of 0.04 mm for prolonged peening times of 48 and 64 s, respectively. The prolonged peening time increased the affected depth of the carburized shaft. As reported by Maleki et al. [14] and Wu et al. [17], a prolonged peening treatment can induce more plastic deformation and increase the microhardness in the subsurface region. In the present study, prolonged peening treatment (up to 64 s) increased not only the microhardness but the affected depth of carburized shaft, as well.

### 3.3. The Effect of Peening Pellets with Different Materials

**Figure 10:**
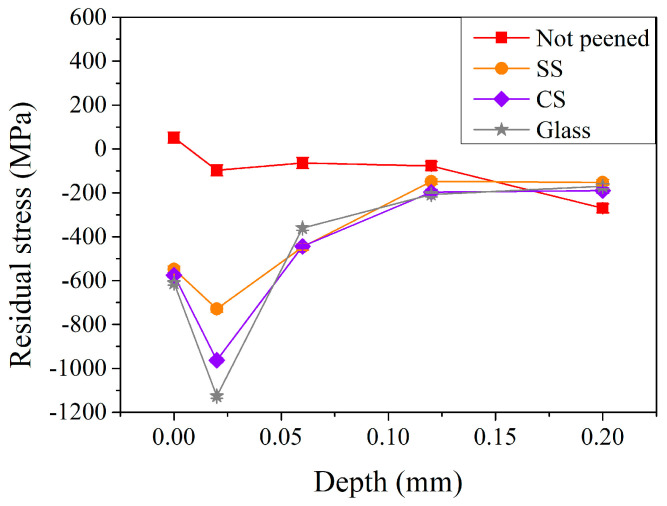
Residual stress profiles of carburized shafts after the peening with different pellet materials.

As demonstrated in the above sections, it can be noted that the optimal parameters for peening treatment were found using stainless-steel pellets with a peening pressure of 5 kg/cm^2^ and a peening time of 64 s. To investigate the effect of peening pellets, another two other types of materials (carbon steel and glass pellets) were chosen to compare to the original stainless-steel pellets, using a peening pressure of 5 kg/cm^2^ but a relatively short peening time of 32 s, based on practical industrial application considerations. Figure 10 shows the residual stress distribution in the subsurface region after the peening with different materials of pellets. Similar behavior could be observed after the shot peening when peening with stainless-steel, carbon steel, and glass pellets. It is interesting to note that the maximum residual stress was −963 MPa when using relatively soft CS pellets (40 HRC, purple curve in Figure 10) compared to that (−779 MPa) of SS (50 HRC, orange curve in Figure 10). The maximum residual stress was further increased to −1126 MPa when treated with glass pellets. All three materials exhibited a hardness lower than that (~60 HRC) of the carburized shaft. Deformation (SS and CS) or fracturing (glass) of peening pellets was expected during processing. The increase in maximum residual stress may be attributed to the irregular shape of peening pellets after the peening treatment. This shows a similar trend to that reported by Lin et al. [10,18]. Though the glass pellets exhibited the highest maximum residual stress, it should be pointed out that they suffered the largest consumption rate during the peening treatment.

**Figure 11:**
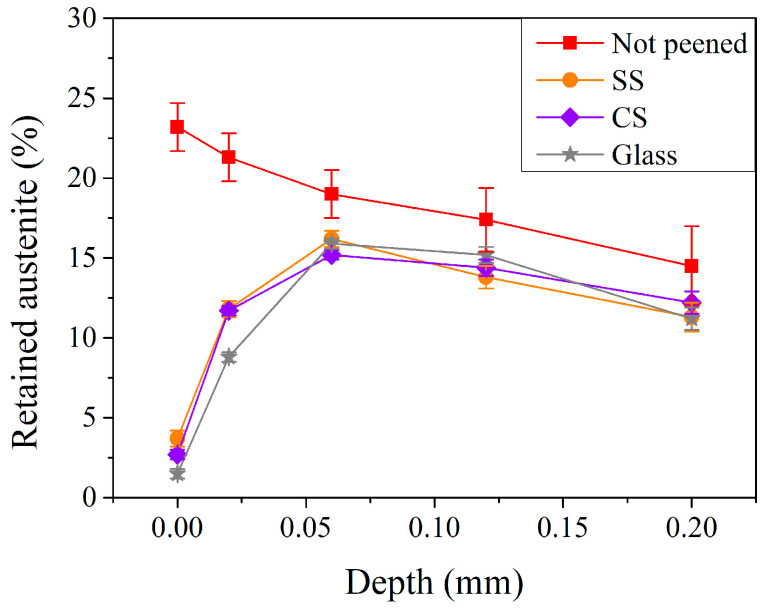
Retained austenite profiles of carburized shafts after the peening with different pellet materials.

The amount of retained austenite in the subsurface region of the carburized shaft after the peening is shown in Figure 11. In contrast to the residual stress, it can be noted that the retained austenite on the surface was 3.7% when using SS peening pellets and decreased to 2.7 and 1.5% when using carbon steel and glass pellets, respectively. After shot peening with CS and SS pellets, the contents of retained austenite within the tested depth (0–0.2 mm) were not obvious. It is, however, interesting to note that, compared to those treated with CS and SS pellets, peening with glass pellets (the grey lines with star symbols) exhibited the lowest retained austenite content in the subsurface region (1.5% and 8.8% at the surface and a depth of 0.02 mm, respectively). This shows a similar behavior to the aforementioned residual stress profiles (Figure 10). This suggests that the increased maximum residual stress can be attributed to the transformation of retained austenite into martensite during the peening treatment. The fractured glass pellets can induce more martensitic transformation in the subsurface region, but the depth is limited to 0.02 mm due to its relatively low impact energy.

**Figure 12:**
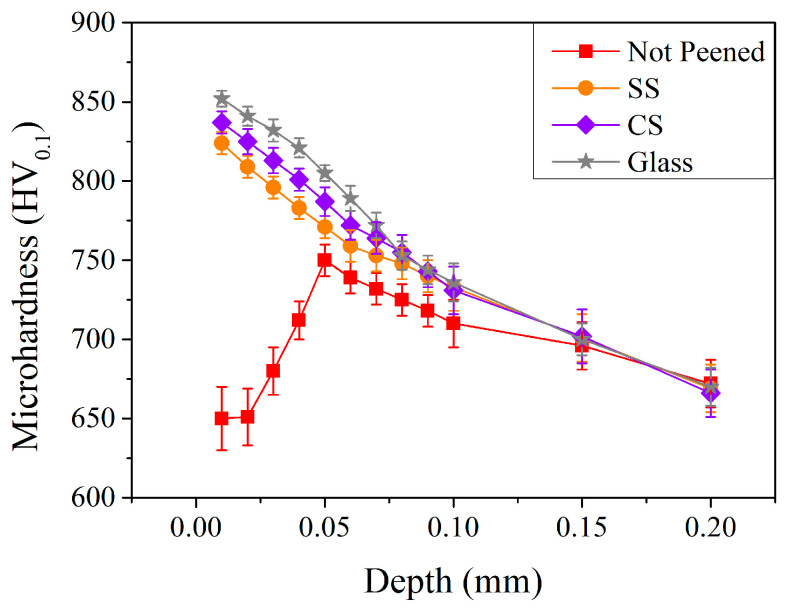
Microhardness profile of carburized shafts after the peening with different pellet materials.

Figure 12 shows the microhardness distribution of the carburized shafts after peening with different pellet materials. Compared to peening with SS pellets (orange curve with circle symbols), peening with relatively soft pellets (CS and glass pellets) resulted in higher microhardness, up to a depth of 0.09 mm. Peening with glass pellets (grey curve with star symbols) resulted in the largest microhardness, at least up to a depth of 0.08 mm. This suggests that not only the microhardness but also the hardened depth caused by glass peening treatment was more evident compared to those treated with CS and SS pellets.

### 3.4. The Surface Roughness of Peening with Different Parameters on the Carburized Shaft

**Figure 13:**
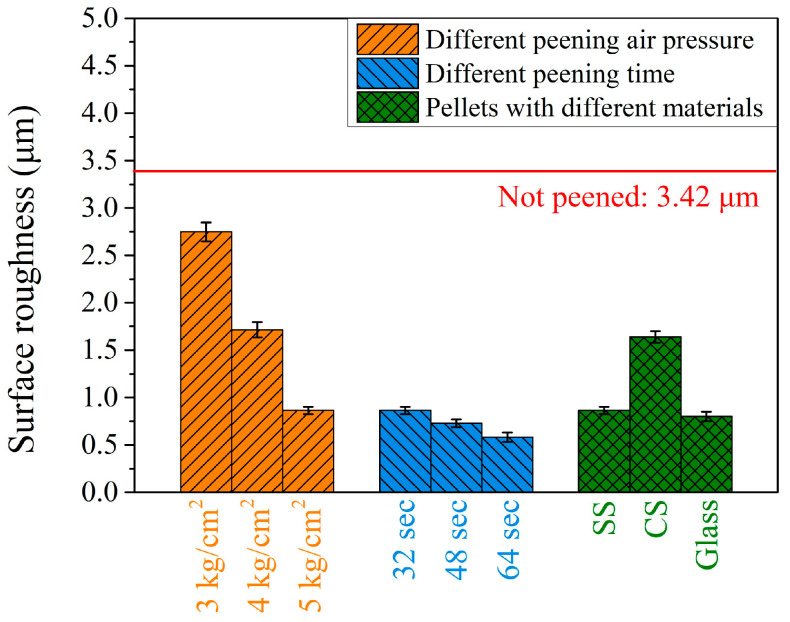
Surface roughness of carburized shafts after peening with various conditions.

The residual stress, the amount of retained austenite, and microhardness in the surface and subsurface regions are important factors governing the lifetime of the carburized shaft that can be prolonged by peening treatment. Another issue affecting the service lifetime of the carburized shaft is the surface roughness after various peening treatments. Figure 13 shows the surface roughness of the carburized shafts after peening with the different peening parameters investigated in the present study. The initial surface roughness was large (3.42 ± 0.13 μm) due to the machining traces, shown in the earlier OM image in Figure 3c. After the peening treatment, the surface roughness decreased significantly. As shown by the three orange bars on the left side in Figure 13, peening with SS pellets using pressures of 3, 4, and 5 kg/cm^2^ resulted in final surface roughness values of 2.75 ± 0.1, 1.71 ± 0.08, and 0.86 ± 0.04 μm, respectively. The higher the peening pressure, the smaller the surface roughness. It can be decreased further by increasing the peening time, as represented by the three blue bars in the middle of Figure 13. The surface roughness values were 0.86 ± 0.04, 0.73 ± 0.04, and 0.58 ± 0.05 μm as a function of peening time (32, 48, and 64 s, respectively). Both the peening pressure and peening time can decrease the surface roughness of the carburized shaft. This showed a similar trend to that reported in the literature [16,17,18]. The surface roughness did not show a monotonic trend when using different peening pellet materials. The surface roughness was 0.86 ± 0.04 μm when peening with SS pellets. It increased to 1.64 ± 0.06 μm using CS pellets and slightly decreased to 0.80 ± 0.05 μm with glass.

Within the limits of the present work, data on the lifetimes of carburized shafts after various peening treatments were not available. Other peening parameters, such as the size (or density) of the pellets, peening angle, and distance, were not covered. The present study revealed that the peening pressure, peening time, and pellet materials affected the residual stress, the amount of retained austenite, and the microhardness on the surface and surface regions. In addition, the surface roughness decreased after peening treatment. Though peening with glass pellets can induce higher microhardness and is expected to result in a longer lifetime, the consumption of glass pellets may be an economic issue in practical applications. Using the CS pellets resulted in slightly better microhardness and residual stress in the subsurface region compared to that treated with SS pellets. Surface roughness after peening, however, was the largest and may not be the optimal choice for practical usage. Using the SS pellets with various peening pressures and peening times can result in various hardening conditions and offer a versatile choice for industrial applications under different considerations. Similar results can be expected not only for another ferrous alloy but some nonferrous alloys, as well.

## 4. Conclusions

In the present study, carburized shafts underwent shot peening treatment using the SS pellets with different peening pressures and durations. Alternative peening materials using the CS and glass pellets were also investigated. The distribution of residual stress, the amount of retained austenite, microhardness, and surface roughness were investigated, and the following conclusions were drawn:

1. The residual tensile stress on the surface of the carburized shaft was converted into a compressive stress, accompanied by a significant decrease in the amount of retained austenite in the subsurface region.
2. The shot peening effect increased with increasing the peening pressure and time. Using the SS pellets with a peening pressure of 5 kg/cm^2^ and a peening time of 64 s resulted in the optimal peening effect. The maximum residual stress reached −779 MPa (at a depth of 0.02 mm), and the microhardness (at the surface) was also the largest (827 HV_0.1_).
3. Peening with different materials can affect the peening results, and glass pellets were more effective than CS and SS pellets. However, the glass pellets suffered the largest consumption rate, while CS pellets resulted in the largest surface roughness. Thus, peening with SS pellets was determined to be the optimal choice for practical industrial application.

## Figures and Tables

**Table 1:** Chemical compositions (wt.%) of the JIS SCM415 steel.

C	Si	Mn	P	S	Cr	Ni	Mo	Cu	Fe.
0.13–0.18	0.15–0.35	0.60–0.85	≤0.03	≤0.03	0.90–1.20	≤0.25	0.15–0.30	≤0.30	Bal.

**Table 2:** Shot peening process parameters.

Pellet Parameters			Peening Parameters	
Material	Hardness	Weight (mg)	Air Pressure (kg/cm^2^)	Time (s)
Stainless Steel	50 HRC	0.90	3	32
Stainless Steel	50 HRC		4	32
Stainless Steel *	50 HRC		5	32
Stainless Steel *	50 HRC	0.90	5	32
Stainless Steel	50 HRC		5	48
Stainless Steel	50 HRC		5	64
Stainless Steel *	50 HRC	0.90	5	32
Carbon Steel	40 HRC	0.89	5	32
Glass	470 HV	0.17	5	32

* The peening parameters were the same for these three tests. Stainless steel and carbon steel are coded as SS and CS, respectively. The pellet size is 0.3 mm for all three pellets. HRC and HV are Rockwell C hardness and Vickers microhardness, respectively.

**Table 3:** The X-ray diffractometer parameters.

Diffractometer Parameters	Specification/Values
Tube type	Cr
Diffraction plane (hkl)	αFe (211)
Bragg angle for diffraction (2θ)	156.5°
Current	1.5 mA
Voltage	30 kV
Exposure time	15 s
Collimator diameter	2 mm
Collimator distance	51 mm

## Data Availability

The original contributions presented in the study are included in the article/Supplementary Material, further inquiries can be directed to the corresponding authors.

## Supplementary Materials

The following supporting information can be downloaded at: https://www.mdpi.com/article/10.3390/ma17164124/s1, Figure S1: Surface images of (a) the original carburized shaft and after shot peening with a pressure of (b) 3, (c) 4, and (d) 5 kg/cm^2^, respectively. Corresponding magnified images in the subsurface region are shown in (e) to (h). Figure S2: X-ray diffraction patterns of the carburized shaft before and after the shot peening treatment. The peaks of martensite for (a) M(200) and (b) M(211) are shown.
