# Supplementary material for: Effects of Shot Peening Pressure, Time, and Material on the Properties of Carburized Steel Shafts
Source: Materials (Basel). 2024 Aug 20;17(16):4124. doi: 10.3390/ma17164124 (PMC11356545; doi:10.3390/ma17164124)
Supplement: Supplementary file 1 [file materials-17-04124-s001.zip › materials-3144395-supplementary.pdf]

Figure S1 shows the magnified surface morphology and microstructure in the subsurface region of the carburized shaft before and after the shot peening treatment. The machining traces before peening disappeared after the shot peening treatment. However, it is still difficult to discriminate the amount of retained austenite.

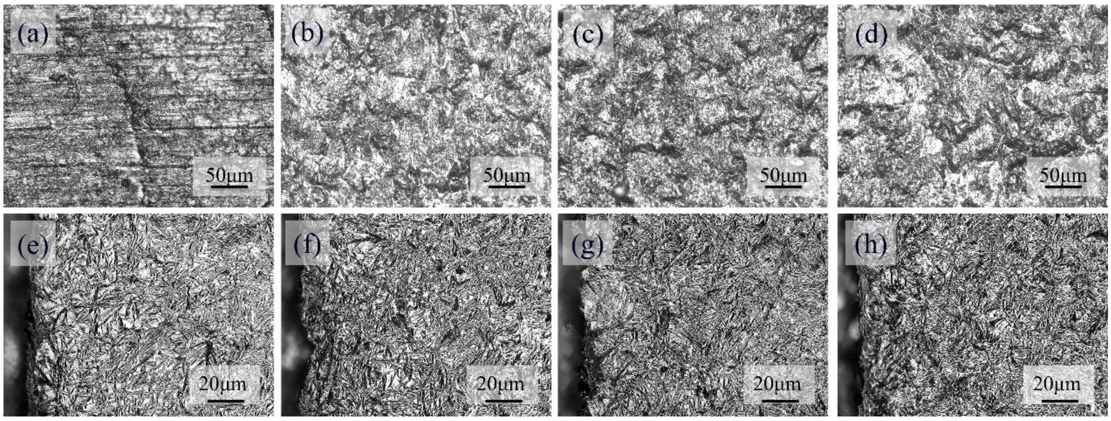

Figure S1: Surface images of (a) the original carburized shaft and after the shot peening treatment with a pressure of (b) 3, (c) 4, and (d) 5 kg/cm^2, respectively. Corresponding magnified images in the subsurface region are shown in (e) to (h).

Figure S2 shows the enlarged XRD patterns of the carburized shaft before and after the shot peening treatment. A slight peak broadening and an increase in the peak intensities of martensite were observed, in the bottom red curve in Figure S2a,b, at 64.59° and 82.04° for M(200) and M(211), respectively.

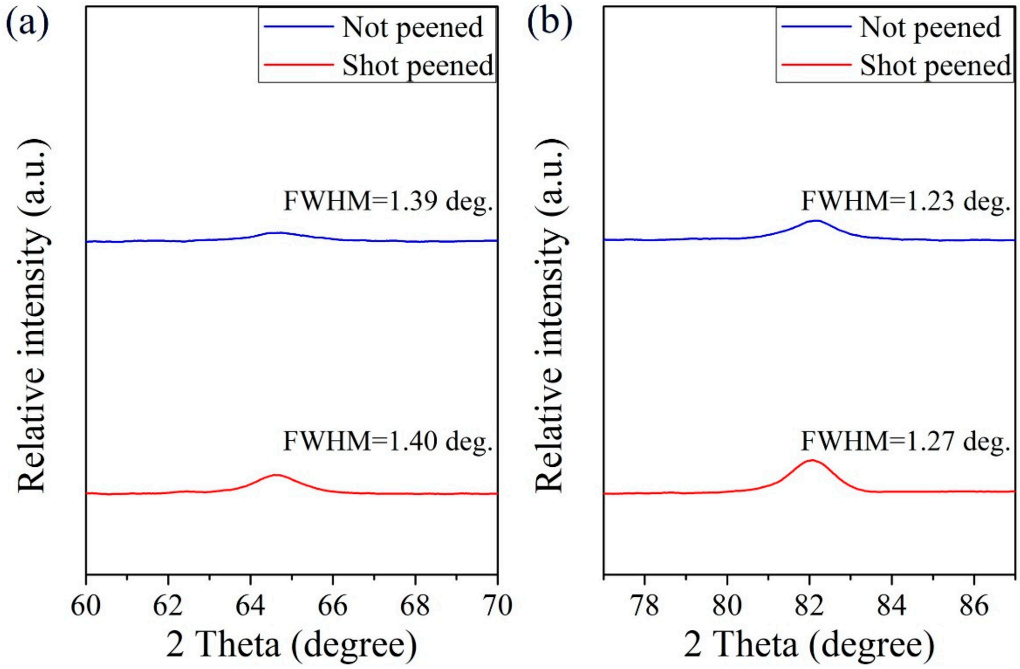

Figure S2: X-ray diffraction patterns of the carburized shaft before and after the shot peening treatment. The peaks of martensite for (a) M(200) and (b) M(211) are shown.
